# How 25(OH)D Levels during Pregnancy Affect Prevalence of Autism in Children: Systematic Review

**DOI:** 10.3390/nu12082311

**Published:** 2020-07-31

**Authors:** Nazlı Uçar, William B. Grant, Isabel Peraita-Costa, María Morales Suárez-Varela

**Affiliations:** 1 Department of Nutrition and Dietetics, Faculty of Health Sciences, Acıbadem University, İçerenköy, Kayışdağı Cd. No:32, Ataşehir/İstanbul 34684, Turkey; nazli.ucar.94@gmail.com; 2 Area of Preventive Medicine and Public Health, Department of Preventive Medicine and Public Health, Food Sciences, Toxicology and Legal Medicine, School of Pharmacy, University de Valencia, Avenida Vicent Andres Estelles s/n, 46100 Valencia, Spain; ipecos@alumni.uv.es; 3 Sunlight, Nutrition, and Health Research Center, P.O. Box 641603, San Francisco, CA 94164-1603, USA; wbgrant@infionline.net; 4 Biomedical Research Center Network on Epidemiology and Public Health (CIBERESP), Institute of Health Carlos III, Avenida Monforte de Lemos, 3-5, Pabellón 11, Planta 0, 28029 Madrid, Spain

**Keywords:** hedging, transaction costs, dynamic programming, risk management, post-decision state variable

## Abstract

Autism spectrum disorder (ASD) is a group of dysfunctions in social interaction, communication, and behaviors. The etiology of ASD is not yet fully understood; however, it consists of the interaction between genetics and the environment. An increasing amount of evidence points to the possibility that gestational and early-childhood vitamin D deficiency may be involved in the etiology of some cases of ASD. Herein, we systematically review the literature for studies on vitamin D status during pregnancy and ASD outcomes. Forty-three studies in the PubMed and 124 studies in EMBASE databases were initially found. After screening, 26 were identified as candidate studies for inclusion. Finally, 14 articles met the inclusion criteria, which originated from nine countries. The studies included 10 original research studies and four review studies conducted between 2012 and 2020. The strength of evidence that vitamin D levels during pregnancy increase the risk of developing autism is very low. This is because the evidence relies exclusively on observational studies that did not equally consider all important confounders and that assessed the indirect relationship between vitamin D as a surrogate for sunlight exposure and autism risk. The findings of this systematic review are consistent with the hypothesis that low vitamin D levels might contribute to the development of autism. However, we must also recognize the possible confusion bias and therefore experimental studies with very large sample sizes, given incidence of autism, that allow us to detect blood levels in pregnant women would be helpful to clarify this point.

## 1. Introduction

As described in the DSM-V, autism spectrum disorder (ASD) is a heterogeneous neurodevelopmental condition affecting an individual’s ability to socialize and communicate [1]. ASD often presents with repetitive movements or behaviors, restricted interests, and abnormality in sensory processing [2]. The prevalence of ASD has risen dramatically over the last several decades, from 1 in 5000 children in 1975 to 1 in 45 children in 2014 in the United States. That trend shows a 700% increase within 40 years [3]. However, the cause of that rapid growth is not fully known. True increase in incidence of autism cannot be ruled out and constitutes an “urgent public health concern” [4,5]. Despite considerable effort to determine the etiological factors of ASD, no single or definitive cause has been identified [6]. The etiology of ASD is unknown, although the interaction of genetic and environmental factors is believed to play a fundamental role in the process [7]. Biological factors possibly associated with autism risk include hereditary risk factors [8]. Despite early evidence of heredity of autism, the largest population-based study of twins with autism is that adaptation rates for fraternal twins are higher than previously reported and that the shared prenatal environment constitutes a greater proportion of the risk of autism in twins [9,10,11]. ASD has been tentatively associated with >440 identified gene variants. Of those cases that can be clearly linked to genetic causes, 7–20% can be accounted for by copy number variants, 5–7% are attributed to single-nucleotide polymorphisms, and <5% are linked to genes involved in rare metabolic disorders [12]. Thus, nearly 70% of cases have a cause that has not been linked to genetics [10]. Lifestyle and environmental factors such as nutrition [13,14], toxic substances [15,16], medications [17,18], maternal infections during pregnancy [19,20], vaccine immunization [21], and stress [22] have been extensively studied. In particular, the factors that cause autism—air pollution, cloudy weather, seasonal factors, migration of dark-skinned immigrants to poleward latitudes, birth order, gestational diabetes, preeclampsia, cesarean delivery, autoimmune disease in the family, and nutrition—are all associated with vitamin D [23,24]. To give an example through studies, gestational vitamin D deficiency was associated with a 2.66-fold higher risk in developing gestational diabetes [25] and in utero exposure to gestational diabetes mellitus, giving the child a 4.4 adjusted odds ratio (aOR) for ASD [26]. Birth via cesarean delivery increases the risk of autism [27], and low maternal vitamin D increases the risk of having to give birth via C-sections up to fourfold [28]. A 2009 study in *Dermato-Endocrinology* examined whether maternal vitamin D deficiency is a risk factor for infantile autism disease (IAD) [29]. In the study using epidemiological data on seasonal variation of birth rates and prevalence of IAD for cohorts born before 1985, the researchers found a strong effective latitudinal (related to wintertime solar UVB radiation) increase in IAD prevalence. Those findings are consistent with the hypothesis that maternal vitamin D deficiency is a risk factor for IAD. Another study examined ASD trends by considering birth data in Denmark, Finland, Norway, Sweden, and Western Australia [30]. The data showed a modest increase in risk for children born in the fall and a modest decrease for children born in the spring. The researchers concluded that the risk was highest for fall births and lowest for spring births, and sunlight levels during critical neurodevelopmental periods explained much of the seasonal trends and are consistent with the hypothesis that a seasonally fluctuating risk factor may influence risk of ASD. In line with several previous studies, an association was apparent between ASD and season of births [31,32,33,34,35]. Moreover, a 2013 ecological study reported that autism prevalence among those aged 6–17 y in 2010 was significantly inversely correlated with solar UVB doses [36]. Those results add to the evidence that vitamin D deficiency may be an important risk factor for autism. In 2008, Cannell proposed the autism–vitamin D theory [37], in which the primary environmental trigger for autism is the lack of vitamin D in pregnancy and early childhood.

Vitamin D metabolism and homeostasis during pregnancy is significantly different than that found outside of pregnancy in order to meet the demands of an optimal intrauterine environment for correct embryonic development and a successful, uncomplicated delivery as well as the immune regulation of the pregnancy [38,39,40]. Pregnancy is characterized by three major adaptations in vitamin D homeostasis that can be seen at both the systemic circulation and placental level; an increase in maternal calcitriol levels, changes in maternal 25(OH)D availability which an affect neonatal 25(OH)D status, and an increase of maternal vitamin D-binding protein concentrations [39]. Calcitriol increases by 2/3 times in the first weeks of pregnancy whereas maternal 25(OH)D crosses the placental barrier and represents the main pool of vitamin D in the fetus and maternal hypovitaminosis D during pregnancy may result in impaired neonatal 25(OH)D status at birth [39]. A limited number of studies have shown changes of vitamin D-binding protein concentrations during pregnancy, with highest concentrations reaching a 40–50% increase compared to non-pregnant women and with levels peaking at the beginning of the third trimester before starting to decrease at term [39]. In regard to immune regulation, the need for immunological tolerance of the mother towards the fetus is a key factor in the changes in vitamin D metabolism that appear during pregnancy [39]. During pregnancy, vitamin D receptor and regulatory metabolic enzymes are expressed in the placenta and decidua, indicating a potential critical point in the immunomodulation at the maternal–fetal interface [39]. Considering these effects, maternal hypovitaminosis D during pregnancy has been associated with pregnancy related disorders and adverse pregnancy outcomes [39].

In recent years, evidence has linked a lack of vitamin D not only to its known effects on calcium and bone metabolism but also to neurocognitive decline [41]. Adequate intake of vitamin D appears essential to global physical and mental health, as suggested by evidence that vitamin D deficiency can be associated with several diseases, such as infections, asthma, inflammatory bowel diseases, obesity, metabolic syndrome, and neuropsychiatric manifestations, including ASD [42]. About 90% of vitamin D is produced in the epidermis from 7-dehydrocholesterol as a reaction to sunlight (solar UVB radiation; 290–315 nm) [43]. Factors that limit the cutaneous production of vitamin D_3_ include higher latitude, covering of skin, lack of outdoor activities, sunscreen use, old age, female sex, and darker skin pigmentation [44]. In an assessment derived from published studies, Holick and colleagues [43] estimated that due mainly to lack of sunlight exposure, approximately 1 billion people worldwide have inadequate vitamin D levels (as defined by level of 25-hydroxyvitamin D [25(OH)D], the primary circulating form of vitamin D in the serum, of <75 nmol/L). In addition to sunlight, another important source of vitamin D is nutrition. People living in regions where sunlight is reduced, such as in northern Europe, need to include in their diets vitamin D-rich foods such as fatty fish or vitamin D-fortified foods [44].

Because it comes from solar UV radiation and exerts endocrine and autoendocrine effects, vitamin D is known as the sunshine hormone [45]. Therefore, the possible mechanisms of action of vitamin D, which is thought to have an important role in preventing and treating ASD, have been reviewed [46,47]. A 2015 study by Huang and colleagues [48] concluded that calcitriol (the active form of vitamin D_3_) can be used to alleviate neuroinflammation (caused mainly by oxidants and toxins). Another mechanism is vitamin D’s effect on serotonin through direct genetic regulation of serotonin rate-limiting enzymes, such as both peripheral tryptophan hydroxylase 1 (TPH1) and central TPH2 [49]. Activated vitamin D hormone (calcitriol) downregulates peripheral TPH1 while upregulating central TPH2, thus explaining the serotonin paradox in ASD in which peripheral serotonin is increased but central serotonin is reduced in those patients [49,50].

Calcitriol protects brain tissue by reducing inflammatory cytokine levels [51]. That increase is strongly associated with cognitive impairment in ASD [52]. Calcitriol also protects the brain by stimulating the production of neurotrophins from several sources. Neurotrophins are chemicals that fight toxic effects [53]. In fact, vitamin D taken orally is pro-prehormone. After ingestion, vitamin D is metabolized by the liver and converted to the 25(OH)D form and then forms calcitriol [(1,25(OH)_2_D]. Calcitriol is a powerful neurosteroid that assists in controlling brain cell growth and acts on vitamin D receptor molecules that appear in most brain cells during the first days of embryo formation [54].

Autistic individuals have abnormalities in immune functions similar to those affected by vitamin D deficiency, such as increased inflammatory cytokine levels [23]. Also, much of the ongoing inflammation in autistic brains is the result of oxidative stress [55,56]. That effect also can be related to vitamin D, which regulates the production of dendritic lymphocytes, in turn reducing the intensity of autoimmune attacks by increasing levels of interleukin 10, an anti-inflammatory cytokine [57].

Herein, we review the recent advances in studying the relationship between vitamin D status during the pregnancy and ASD in the outcome to try to identify the potential role of vitamin D in the etiology of ASD.

## 2. Materials and Methods

To report this systematic review, we followed the guidelines specified in PRISMA (Preferred Reporting Items for Systematic Reviews and Meta-Analyses) [58,59].

### 2.1. Literature Search

**Figure 1:**
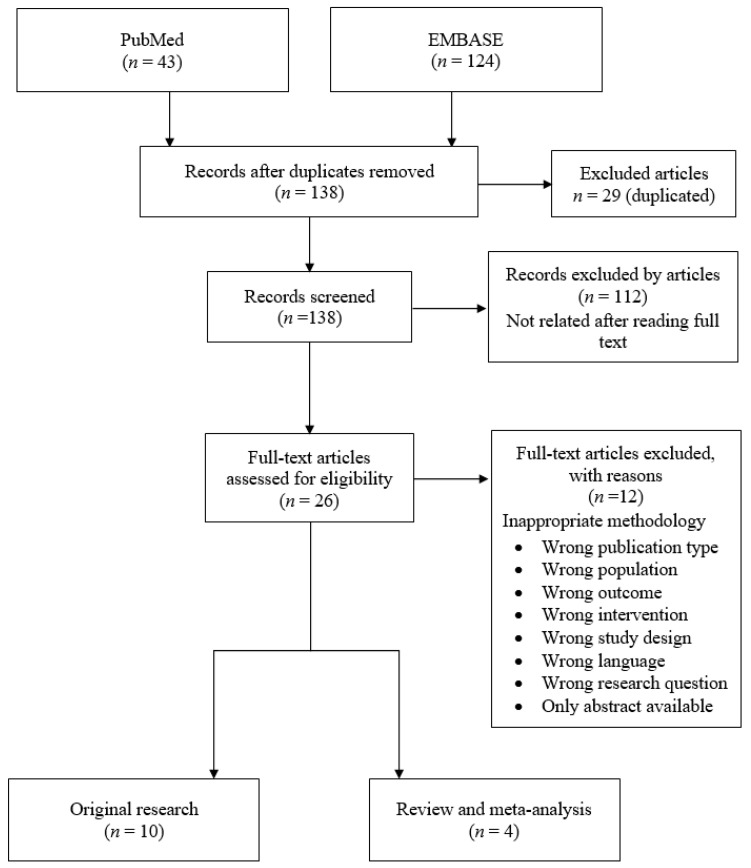
Search strategy.

We searched the literature to identify publications eligible for inclusion in the review in the PubMed and EMBASE databases. The literature search strategy used included the keywords “pregnancy,” “gestation,” “vitamin D,” “autism,” and “autism spectrum disorder.” The search was limited to human subjects and English language articles published between 2010 and January 2020. Forty-three studies came from PubMed and 124 studies from EMBASE, for a total of 167 articles identified and retrieved. An initial screening identified 26 candidate studies. Figure 1 shows a PRISMA flowchart indicating the search strategy followed. After articles were assessed for eligibility, they were then identified and the first screening of the articles was performed using the information available in the abstract and result section of each study.

### 2.2. Study Inclusion/Exclusion Criteria and Data Extraction

We included randomized and nonrandomized controlled trials, prospective cohort studies, case-control studies, and systematic reviews on longitudinal studies that investigated how vitamin D levels during pregnancy affected the prevalence of autism in children. The selection criteria included the following:

1. Human study of vitamin D supplementation during pregnancy or vitamin D status of pregnancy or vitamin D status of ASD children;
2. Available information of circulation concentration of 25(OH)D in maternal blood during pregnancy or in newborn blood at birth;
3. Original research article and review in humans (abstracts, case reports, ecological studies, and comments were excluded);
4. Outcomes including incidence of autism, measures of autism, severity, measures of neurophysiological aspects of autism, or expression of autism-related genes and vitamin D during pregnancy; and
5. Article written in English
  - Any study between 2010 and January 2020.

Any study that involved children with any other disease that could affect serum vitamin D levels was excluded. Only publications meeting all criteria were included in this analysis. After a full assessment of the potentially relevant studies, 14 were proposed to be included in this systematic review.

### 2.3. Study Quality

We evaluated the methodological quality (risk of bias) of studies by using the Newcastle–Ottawa Scale (NOS) for observational studies [60,61]. The NOS criteria included (1) subject selection, 0–4; (2) comparability of subject, 0–2; and (3) clinical outcome, 0–3. Total NOS scores range from 0 (lowest) to 9 (highest). On the basis of NOS scores, studies can be divided into low quality (0–6) and high quality (7–9). Two evaluators conducted the NOS assessment independently. Any discrepancy on NOS scores of potential studies was resolved through discussion or consultation with a third evaluator.

For a more detailed evaluation of the selected articles, the scale proposed by the Scottish Intercollegiate Guidelines Network (SIGN) was used, establishing levels of evidence (Table 1) and recommendations (Table 2) [62]. The SIGN system yields a detailed assessment of the degree of scientific evidence. Epistemological strength is the way the articles are classified. Only the strongest evidence gives way to strong recommendations, whereas weaker evidence can give rise only to weak recommendations. The SIGN system emphasizes that studies with a high rate of bias may lead to biased results, and the bias level of the studies is determined with that system. The risk of bias and study design are used to allow assessment of the level of evidence and the quality of scientific evidence provided. When the numbers 1, 2, 3, and 4 are used to grade the study, ++, +, and −- signs are used to indicate the risk of bias. On the basis of that assessment of the quality of evidence in the articles, they are classified best to worst according to grades A, B, C, and D. That scale rating is constructed on the principles of evidence-based medicine—an approach that ensures the use of the most up-to-date, reliable, and scientifically solid evidence available in making decisions about a particular situation being studied [63].

We also focused on the internal validity of the studies analyzed. The factors that influence internal validity that were considered are a sample size that was large enough and that inclusion and assessment criteria were specified.

## 3. Results

### 3.1. Study Characteristics

The chosen studies were analyzed according to study design, location, participant, sample size, target population, and major findings. Table 3 and Table 4 summarize the characteristics of the studies.

In total, 167 articles were initially assessed in this study. After removing 29 duplicated articles, we screened 138 by their abstracts. Twenty-six articles were retrieved for full-text assessment. Finally, 14 articles from nine countries met the inclusion criteria [30,31,32,64,65,66,67,68,69,70,71,72,73,74]. The studies included 10 original research and 4 review studies conducted between 2012 and 2020.

The original research studies used data from five countries: China [31,32], Sweden [30,64,65], the Netherlands [66,67], Spain [68], and Australia [69]. Data from one study were obtained from neighboring states, across the United States, Canada, and Israel [70]. The review research studies included data from the United States, Australia, Sweden, China, United Kingdom, Spain, Denmark, the Netherlands, Vietnam, the Republic of Seychelles, India, Greece, Egypt, Brazil, Turkey, Qatar, Saudi Arabia, Asia, and Europe [71,72,73,74].

### 3.2. Original Research Studies

Chen and colleagues [31] have shown that mothers in the autistic group had significantly lower maternal serum levels of 25(OH)D than in neurotypical groups, with 55.9% and 29.4% being vitamin D deficient, respectively. Lower first-trimester maternal serum levels of 25(OH)D were associated with a significantly increased risk of developing autism in offspring (*p* < 0.001). Fernell and colleagues [65] analyzed 25(OH)D in 58 Sweden-born sibling pairs, in which one child had ASD and the other did not. The collapsed group of children with ASD had significantly lower vitamin D levels than their siblings. The findings suggest that low prenatal vitamin D may act as a risk factor for ASD (*p* = 0.013). Stubb and colleagues [70] published an open-label prospective study in 2016, prescribing vitamin D during pregnancy to the expectant mothers of children with autism at a dose of 5000 IU/day. The newborn children were monitored for 3 years, during which they were assessed for autism at 18 and 36 months of age. The final outcome was that 1 of 19 (5%) developed autism in contrast to the overall recurrence rate of approximately 20%, which is evident across the literature (*n* = 20).

Vinkhuyzen and colleagues [66] in their study based on a Rotterdam birth cohort (*n* = 4334), found that individuals in the 25(OH)D-deficient group at midgestation had more than a twofold increased risk of ASD than the 25(OH)D-sufficient group (*p* = 0.03). Also, in another study conducted by Vinkhuyzen and colleagues [67] in 2018, they aimed to determine the relationship between vitamin D deficiency in pregnancy and Social Responsiveness Scale (SRS) status of their children (*n* = 4229). 25(OH)D concentration was measured in serum and was measured from two samples with the first sample taken at 20.6 weeks and the second sample collected at birth, from neonatal cord blood. The 25(OH)D-deficient group had significantly higher (*p* < 0.001) (more abnormal) SRS scores than the 25(OH)D-sufficient group [25(OH)D > 50 nmol/L]. Those study results serve to clarify the relationship between maternal vitamin D deficiency and offspring risk of ASD with and without intellectual disability.

The Stockholm Youth Cohort is a registry-based total population study (*N* = 509,639) [64] in which maternal vitamin D deficiency, defined as a lifetime-recorded diagnosis of unspecified vitamin D deficiency (ICD-10

E55.9 or ICD-9 268.9) corresponding to a serum 25-hydroxyvitamin D level of less than 25 nmol/L, was associated with offspring risk of ASD with, but not without, intellectual disability. In a 2019 study, Lee and colleagues [30] found that neonatal 25(OH)D <25 nmol/L was associated with 1.33 times higher odds of ASD (95% confidence interval (CI), 1.02 to 1.75) than for levels of 25(OH)D ≥50 nmol/L. In Nordic-born mothers, maternal 25(OH)D insufficiency (25 to <50 nmol/L) at ~11 weeks’ gestation was associated with 1.58 times higher odds of ASD (95% CI, 1.00 to 2.49) than for 25(OH)D sufficiency (≥50 nmol/L). Wu and colleagues [32] analyzed the concentration of 25(OH)D_3_ in children with ASD, and controls were assessed from neonatal dried blood samples. The median 25(OH)D_3_ level was significantly (*p* < 0.0001) lower in children with ASD than for controls. As a result, neonatal vitamin D status was significantly associated with the risk of ASDs and intellectual disability.

A 2019 study [68] clearly showed that vitamin D deficiency during critical periods of pregnancy development could lead to persistent brain alterations in the fetus. Maternal plasma vitamin D_3_ was measured in pregnancy through a single maternal blood sample at a mean of 13.3 weeks of gestation, with findings that per each 10-ng/mL increment of maternal vitamin D_3_, children obtained higher social competence scores at 5 years old (coefficient = 0.77; 95% CI, 0.19 to 1.35). Also, in a cohort study by Whitehouse and colleagues [69], offspring of mothers with low 25(OH)D concentrations (<49 nmol/L) at 18 weeks of gestation showed increased risk for “high” scores (≥2 standard deviations above the mean) on the Attention Switching subscale.

### 3.3. Review and Meta-Analysis Studies

Diverse studies have investigated the impact of prenatal exposure to vitamin D levels on brain development and autism. A systematic review and meta-analysis published in 2019 [71] summarized evidence of the association between 25(OH)D levels in maternal blood in pregnancy or newborn blood at birth and neurodevelopmental outcomes. Those outcomes included cognition, psychomotor performance, language development, behavioral difficulties, attention deficit–hyperactivity disorder (ADHD), and autistic traits. That meta-analysis offers supporting evidence that increased prenatal exposure to 25(OH)D levels is associated with improved cognitive development and reduced risk of ADHD and autism-related traits later in life. Furthermore, an alternative review [72] showed that depending on the timing of the exposure to low vitamin D status, different brain areas might be affected, possibly causing different neurodevelopment and cognitive outcomes in infants. In agreement with other studies, Kočovská and colleagues and Wang and colleagues [73,74] showed that decreased vitamin D levels during pregnancy and decreased exposure to solar UVB might increase the risk for ASD.

### 3.4. Summary of Findings

Vitamin D plays many roles in various processes in the human body. Supporting findings from the past 15 years have come from animal studies, human molecular, cellular and physiological, genetic, and neurodevelopmental studies. And vitamin D deficiency during pregnancy or early childhood has been suggested as a possible environmental risk factor for ASD.

Our literature review identified many observational studies, but few intervention trials have been conducted that investigate the relationship between vitamin D level and ASD during pregnancy. The strength of evidence that vitamin D levels during pregnancy increase the risk of developing autism is very low. That evidence exclusively relies on observational studies that did not equally consider all important confounders and that assessed the indirect relationship between vitamin D as a surrogate for sunlight exposure and autism risk.

Despite the limited and inconclusive results of meta-analyses, research has established that early exposure to inadequate vitamin D may interact with other factors and contribute to the etiology of autism, and individuals with ASD may be a population at risk of vitamin D deficiency/inadequacy. Therefore, evidence-based clinical recommendations are needed to prevent ASD and manage ASD symptoms. Until better data are available, vitamin D is worth considering as a potential preventive measure for ASD.

## 4. Discussion

ASD, a complex, heterogeneous neurodevelopmental disorder, had an estimated prevalence of ~1% in children in 2017 [75]. ASD is associated with biological and environmental factors. Epigenetic mechanisms have detected that gene–environment interactions are important in mediating risk [76,77].

ASD and low vitamin D are commonly interrelated, and children with ASD also have vitamin D deficiencies. Vitamin D deficiency affects ~1 billion people globally and is a potential environmental risk factor for ASD [43]. Epidemiological studies and data obtained in humans have yielded evidence that a mother’s diet during pregnancy plays a vital role in the development of the neural circuitry that regulates behavior, thus determining persistent behavioral effects in the offspring [78]. Generally, vitamin D concentration during pregnancy is associated with a higher or lower incidence of ASD or autistic traits in offspring [79]. A 2013 report [36] emphasized that people with low activity of vitamin D enzymes and people with maternal or early childhood vitamin D deficiency would have low activity in the vitamin D system, which is important for brain development. In addition, through several feasible mechanisms, vitamin D can help children with autism.

Considering whether prenatal vitamin D deficiency is associated with the development of ASD seems reasonable. The findings suggest that autistic children have lover 25(OH) D levels than their unaffected siblings [67,80]. That finding emphasizes that more randomized controlled trials are needed. But until new studies are completed, it seems prudent to maximize vitamin D levels in pregnant and lactating women, infants, and young children, as the Endocrine Society recommends, aiming for levels found in humans living in a sun-rich environment (46 ng/mL) [67,80].

Furthermore, nutritional quality control in the mother’s diet during pregnancy is a potentially effective strategy to prevent autism in the offspring. Nutritional deficiencies can be prevented by using safe and easily accessible supplements. That effect is seen in the results of our review, which show that using the right amount of vitamin D during pregnancy may reduce the risk of ASD [81]. Currently it is suggested that all pregnant women maintain a circulating 25(OH)D concentration of at least 40 ng/mL for the first 16 weeks of gestation [82] and to achieve this, intakes of at least 4000 IU/d vitamin D3 would be necessary [83,84]. Supplementation could be especially important in women with children diagnosed with ASD as previous studies have suggested that supplementation could reduce the recurrence rate of autism in siblings [65,70,85]. However, while the results of these studies are promising, these are preliminary studies with small sample sizes. Therefore, in order to adequately inform recommendations for mothers with children diagnosed with ASD, further research in larger samples is needed to confirm the results as well as to determine appropriate dosage.

By contrast, in a study that included maternal vitamin D levels, no relationship was apparent between maternal vitamin D level and autism. That study reported that no relationship existed between maternal vitamin D levels and autism in children of both white and Somali mothers [86], but fewer than 20 mothers were included in each group and analyses were performed several years after birth. A review in January 2020 concluded that vitamin D deficiency in the data available now does not adequately support the hypothesis that it may contribute to the etiology of ASD [87].

Future epidemiological studies should include prenatal or neonatal vitamin D levels and have enough statistical power to show convincing evidence of a relationship between low neonatal vitamin D status and the risk of autism.

### Strengths and Limitations of This Review

The clinical trials included in our analysis show significant differences in sample size, use of reinforcements, and follow-up. In addition, researchers may not be able to access all publications on the relationship between vitamin D and autism during pregnancy because the area of analysis is limited to studies published in English and available through the PubMed and EMBASE databases. However, the main outcome measured was common in all the studies and included the risk/incidence of autism/ASD in offspring.

## 5. Conclusions

The findings of this systematic review are consistent with the hypothesis that low vitamin D levels might contribute to the development of autism. However, we also must recognize the possibility of confusion bias because a diet with adequate levels of vitamin D is usually also adequate in other micronutrients. Experimental studies that allow us to detect blood levels in pregnant women would therefore be helpful to clarify that point. However, we must bear in mind that doing so would require a design with very large sample sizes given the incidence of autism. Further studies examining the relationship between vitamin D and autism risk are needed.

## Figures and Tables

**Table 1:** Levels of evidence.

Grade	Risk of Bias	Description
1	1++	High-quality meta-analyses, systematic reviews of RCTs, or RCTs with very low risk of bias
	1+	Well-conducted meta-analyses, systematic reviews of RCTs, or RCTs with low risk of bias
	1−−	Meta-analyses, systematic reviews, or RCTs with high risk of bias
2	2++	High-quality systematic reviews of case-control or cohort studies; high-quality case-control or cohort studies with very low risk of confounding or bias and high probability of casual relationship
	2+	Well-conducted case-control or cohort studies with low risk of confounding or bias and moderate probability of casual relationship
	2−−	Case-control or cohort studies with high risk of confounding or bias and significant risk of noncausal relationship
3		Nonanalytic studies, e.g., case reports, case series
4		Expert opinion

RCT, randomized controlled trial.

**Table 2:** Grades of recommendation.

Grade	Description
A	At least one meta-analysis, systematic review, or RCT rated 1++, and directly applicable to target population A body of evidence consisting principally of studies rated 1+, directly applicable to the target population, and demonstrating overall consistency of results
B	A body of evidence including studies rated 2++, directly applicable to target population, and demonstrating overall consistency of results Extrapolated evidence from studies rated 1++ or 1+
C	A body of evidence including studies rated 2+, directly applicable to the target population, and demonstrating overall consistency of results Extrapolated evidence from studies rated 2++
D	Evidence level 3 or 4Extrapolated evidence from studies rated 2+

**Table 3:** Original research studies on 25-hydroxyvitamin D (25(OH)D) during pregnancy and autism.

Ref.	Location	Study Design	Period of Study	Participant	Sample Size	Target Pop.	Exposure Assessment Vitamin D	Major Findings	LE	GR	NOS
Wu et al. (2018) [32]	Beijing, China	Case-control	2008–2010	Newborn screening in Beijing (NBSIB)	*n* = 32,912 (*n* = 310 ASD and *n* = 1240 controls)	Children monitored from birth to age 3 years	Tiny sample of blood; liquid chromatography–tandem mass spectrometry	Neonatal vitamin D status was significantly (*p* < 0.0001) associated with risk of ASDs and intellectual disability. Compared with the fourth quartiles, the RR of ASDs was significantly increased for neonates in each of the three lower quartiles of the distribution of 25(OH)D3, and increased risk of ASDs by 260% (RR for lowest quartile, 3.6; 95% CI, 1.8 to 7.2; *p* < 0.001), 150% (RR for second quartile: 2.5; 95% CI, 1.4 to 3.5; *p* = 0.024), and 90% (RR for third quartile: 1.9; 95% CI, 1.1 to 3.3; *p* = 0.08), respectively.	2++	B	8
B.K. Lee et al. (2019) [30]	Stockholm County, Sweden	Cohort	Participants born in Sweden, 1996–2000	Stockholm Youth Cohort (*n* = 98,597)	Maternal sample consisted of 449 ASD cases and 574 controls, the neonatal sample: 1399 ASD cases and 1607 controls; and the paired maternal–neonatal sample: 340 ASD cases and 426 controls	Cohort of all children aged 0–17 years living in Stockholm County	Maternal sample examining prenatal 25(OH)D in maternal sera; neonatal sample examining neonatal 25(OH)D in dried blood spots taken soon after birth; neonatal sibling sample examining neonatal 25(OH)D in cases matched to unaffected siblings; and paired maternal–neonatal sample examining both maternal and neonatal 25(OH)D. 25(OH)D was measured using a tandem mass spectrometry assay.	In Nordic-born mothers, maternal 25(OH)D insufficiency (25–<50 nmol/L) at ~11 weeks’ gestation was associated with 1.58 times higher odds of ASD (95% CI, 1.00 to 2.49) than 25(OH)D sufficiency (≥50 nmol/L). Neonatal 25(OH)D <25 nmol/L was associated with 1.33 times higher odds of ASD (95% CI, 1.02 to 1.75) than 25(OH)D ≥50 nmol/L.	2++	B	8
*M.L. Vicente et al. (2019)* [68]	Spain: Asturias, Gipuzkoa, Menorca, Sabadell, Valencia	Cohort	Feb. 1997–Sept. 1998, Menorca; Nov. 2003–Feb. 2008, other regions	Embedded in the Infancia y Medio Ambiente (INMA) Project, a population-based birth cohort based in regions across Spain	*N* = 3216; *n* = 2107 (mother–child pairs)	10–13 weeks of gestation and their child’s neurodevelopmental assessment at 5, 8, 14, and 18 years old	Maternal blood and child plasma concentrations of 25(OH)D3 by high-performance liquid chromatography method using a Bio-Rad kit according to Clinical and Laboratory Standard Institute protocols	Per each 10-ng/mL increment of maternal vitamin D_3_, children obtained higher social competence scores (β-coefficient = 0.77; 95% CI, 0.19 to 1.35) at 5 y old	2++	B	8

25(OH)D, 25-hydroxyvitamin D; 95% CI, 95% confidence interval; ASD, autism spectrum disorder; GR, grade of recommendation; LE, level of evidence; NOS, Newcastle–Ottawa Scale; RR, relative risk.

**Table 4:** Review and meta-analysis studies on vitamin D during pregnancy and autism 3.2. Study Design and Population.

Ref.	Design	Studies Included			Location and Year	Participants	Sample Size (Case with ASD/Control)	ASD Diagnosis	Findings	Major Findings	LE	GR
		Common	Different	Design								
E. Kocovska et al. (2012) [73]	Review	Humble et al. (2010)		No controls, cross-sectional	Sweden	European adults psychiatric disorder incl. autism	*N* = 117 (117/0)		Significant difference (*p* < 0.005) The median (25th–75th percentile) is in autistic patients, 31.5 ng/mL (23–39); and in all patients, 45 ng/mL (31–60)	Vitamin D deficiency in pregnancy or early childhood may be an environmental trigger for ASD in people genetically susceptible to autism.	2++	B
		Meguid et al. (2010)		Case-control, cross-sectional	Egypt	Children with or without autism	*n* = 112 (70/42)	DSM-IV for clinical diagnosis	Significant difference (*p* < 0.005) In autistic children, 28.5 ng/mL Controls: typically developing children 40.1 ng/mL In addition, children with autism had significantly lower 25(OH)D (*p* < 0.00001) and 1,25(OH)(2)D (*p* < 0.005) as well as lower calcium (*p* < 0.0001) serum values than the controls.			
			Molloy et al. (2010)	Case-control, cross-sectional	USA	White male children aged 4–8 years with ASD	*n* = 89 (49/40)	DSM-IV/ADOS	No significant difference (*p* = 0.4) Cases: 20 ng/mL All Participants <31 ng/mL.			
			Fernell et al. (2010)	Case-control, cross-sectional	Sweden	Mothers of Somali or Swedish origin with a child with or without autism	*n* = 80 (40/40)		Significant difference Somali vs. Swedish mothers Cases: Somali mothers with autism child 6.7 ng/mL; Swedish mothers with autistic child 24.8 ng/mL Controls: Somali mothers with nonautistic child 9.6 ng/mL; Swedish mothers with nonautistic child 20.7 ng/mL.			
H. Mazahery et al. (2016) [72]	Review	Humble et al. (2010)		No controls, cross-sectional	Sweden	European adults psychiatric disorder incl. autism	*n* = 117 (117/0)		Significant difference (*p* < 0.005) In autistic patients, 31.5 ng/mL (23–39) In all patients, 45 ng/mL (31–60).	Low vitamin D status in utero, postnatal, and early childhood might affect different brain regions, and that situation might cause different neurodevelopmental and cognitive outcomes in infants.	2++	B
		Stubbs et al. (2016)		Prospective cohort (uncontrolled)	USA	Pregnant mothers of autistic children aged 18 months–3 years	*n* = 20 (no control)	MCHAT and PDDBI	Although autism recurrence rate reported in the literature is 20%, recurrence rate of autism in newborns was 5%.			
		Saad et al. (2015)		Open-label intervention	Egypt	Autistic children with 25(OH)D <75 nmol/L	*n* = 222 (122/100)	DSM-IV/CARS, ABC ^2^	13% (*n* = 16): normal serum 25(OH)D concentration 57% (*n* = 70): vitamin D deficiency, 30% (*n* = 36): vitamin D insufficiency Mean 25(OH)D levels in patients with severe autism were significantly lower than patients with mild or moderate autism (*p* < 0.0001). Serum 25(OH)D levels showed significant negative correlation with CARS scores (*r* = −0.502, *p* < 0.0001).			
			Azzam HME et al. (2015)	RCT	Egypt; from January 2009 until June 2010	Children with ASD	*n* = 21 (21/0)	DSM-IV	No significant difference (three groups: 2.7 ± 1.9 y for vitamin D–supplemented group, 5.8 ± 2.9 y for unsupplemented autistic group, 5 ± 1.8 y for neurotypical control group) Mean presupplementation CARS scores for the two autistic groups were 33.9 ± 2.9 and 33 ± 3 (*p* > 0.05). Mean VABS was 51.4 ± 16 and 55.7 ± 20 (*p* > 0.05).			
			Ucuz İİ et al. (2015)	Open-label intervention	Turkey	Toddlers aged 2–5 years with developmental delay without and with ASD	*n* = 66; 25(OH)D <50 nmol/L (*n* = 11 cases) and ≥50 nmol/L (*n* =10 controls)	ABC ^1^ Denver II	No significant difference between groups with normal and low vitamin D levels in terms of ADSI, Denver II, CBCL, and ABC scores, and NGF and GDNF levels (*p* > 0.05).			
			Jia F et al. (2015)	Case	China	32-month-old male toddler with ASD		ABC ^1^ CARS	Some autism symptoms improved significantly after vitamin D_3_ supplementation.			
			Feng J et al. (2016)	Open-label intervention	China	Children with ASD	*n* = 500 (215/285)	DSM-IV/ADOS	Vitamin D3 may play an important role in etiology of ASD. Serum levels of 25(OH)D were significantly lower in ASD children than control group (*p* < 0.05).			
T. Wang et al. (2016) [74]	Systematic review and meta-analysis	Fernell et al. (2015)		Sibling–control	Sweden, 2005–2008	Gothenburg catchment area group and Stockholm Somali group; children aged 4+ years	*n* = 116 (58/58)	Clinical report; ASD diagnosis	Collapsed group of children with ASD had significantly lower vitamin D levels (Mean = 24.0 nM, SD = 19.6) than siblings (Mean = 31.9 nM, SD = 27.7) (*p* = 0.013).	Decreased vitamin D levels in patients, decreased maternal vitamin D levels during pregnancy, and decreased exposure to solar UVB might increase the risk for ASD.	2++	B
		Meguid et al. (2010)		Case-control, cross-sectional	Egypt	Children with or without autism	*n* = 112 (70/42)	DSM-IV for clinical diagnosis	Significant difference (*p* < 0.005) In autistic children, 28.5 ng/mL Controls: typically developing children 40.1 ng/mL In addition, children with autism had significantly lower 25(OH)D (*p* < 0.00001) and 1,25(OH)(2)D (*p* < 0.005) as well as lower calcium (*p* < 0.0001) serum values than the controls.			
		Saad et al. (2015)		Open-label intervention	Egypt	Children with ASD	*n* = 222 (122/100)	DSM-IV/CARS, ABC ^2^	13% (*n* = 16): normal serum 25(OH)D concentration 57% (*n* = 70): vitamin D deficiency, 30% (*n* = 36): vitamin D insufficiency Mean 25(OH)D levels in patients with severe autism were significantly lower than patients with mild or moderate autism (*p* < 0.0001). Serum 25(OH)D levels showed significant negative correlation with CARS scores (*r* = −0.502, *p* < 0.0001).			
			Adams et al. (2011)	Randomized, double-blind, placebo-controlled	U.S.	Two groups, an Arizona group aged 5–16 years and a National group aged 3–60 y; children and adults with autism	*n* = 141 (53/88)	DSM-V	No significant differences between ASD and control group in vitamin D_3_ [25(OH)D in plasma; *p* = 0.04].			
			Mostafa et al. (2012)	Cross-sectional	Saudi Arabia, April–September 2012	Children aged 5–12 years with and without ASD	*n* = 80 (50/30)	DSM-IV	Autistic children had significantly lower serum levels of 25(OH)D than healthy children (*p* < 0.001). In addition, serum 25(OH)D levels had significant negative correlations with CARS (*p* < 0.001) and serum levels of anti-MAG autoantibodies (*p* < 0.001).			
			Neumeyer et al. (2013)	Cohort	U.S.	Boys aged 8–14 years with and without ASD	*n* = 37 (18/19)	DSM-IV	Serum 25(OH)D levels were lower in boys with ASD than in controls (*p* = 0.05).			
			Uğur et al. (2014)	Case-control	Turkey	ASD (recruited for the study); non-ASD healthy controls	*n* = 108 (54/54)	DSM-IV	No difference between ASD and healthy controls.			
			Gong et al. (2014)	Case-control	China, January–December 2012	Children with and without ASD; consecutive patients with ASD admitted to Dept. of Neurology	*n* = 96 (48/48)	DSM-IV, CARS (all cases)	Significant negative association between serum 25(OH)D levels and CARS scores (*p* = 0.000).			
			Bener et al. (2014)	Case-control	Qatar, June 2011–May 2013	Children aged 3–8 years; children with and without ASD	*n* = 508 (254/254)	DSM-IV	Significant difference found in mean values of vitamin D between autism (18.39 ± 8.2 ng/mL with median 18) and versus control children (21.59 ± 8.4 ng/mL) (*p* < 0.0001) and with median 21 (*p* = 0.004).			
			DU et al. (2015)	Cohort	China	Children with and without ASD	*n* = 226 (117/109)		Lower 25(OH)D in ASD group (*p* < 0.01).			
			Tostes et al. (2012)	Cohort	Brazil	Children with and without ASD	*n* = 48 (24/24)	DSM-IV	Serum levels of 25(OH)D were lower in children with autism (26.48 ± 3.48 ng/mL) than in typically developing subjects (40.52 ± 3.13 ng/mL) (*p* < 0.001).			
A.M. Garcia-Serna and E. Morales (2019) [71]	Systematic review and meta-analysis	Stubbs et al. (2016)		Prospective cohort (uncontrolled)	U.S.	Pregnant mothers of autistic children; 18 months–3 years	*n* = 20 (no control)	MCHAT and PDDBI	Although the autism recurrence rate reported in the literature is 20%, the recurrence rate of autism in newborns was 5%.	Meta-analysis showed higher levels of prenatal 25(OH)D to be associated with a lower risk of autistic traits.	2++	B
		Fernell et al. (2015)		Sibling–control	Sweden, 2005–2008	Gothenburg catchment area group and Stockholm Somali group; children aged 4+ years	*n* = 116 (58/58)	Clinical report; ASD	Collapsed group of children with ASD had significantly lower vitamin D levels (M = 24.0 nM, SD = 19.6) than their siblings (M = 31.9 nM, SD = 27.7) (*p* = 0.013).			
			Morales et al. (2012)	Cohort	Spain, 2003–2008	Mothers (13 weeks) 25(OH)D; children aged 14 months	*n* = 1820	Psychologist report; BSID	Infants of mothers with 25(OH)D3 concentrations in pregnancy >30 ng/mL showed higher mental score (β = 2.60; 95% CI, 0.63 to 4.56) and higher psychomotor score (β = 2.32; 95% CI, 0.36 to 4.28) than those of mothers with 25(OH)D_3_ concentrations <20 ng/mL. The median plasma value of 25(OH)D (3) in pregnancy was 29.6 ng/mL (interquartile range, 21.8–37.3).			
			Morales et al. (2015)	Cohort	Spain; 1997–2008	Mothers (13 weeks) 25(OH)D; children aged 4–8 years	*n* = 1650	DSM-IV	Total ADHD-like symptoms in children decreased by 11% per 10-ng/mL increment of maternal 25(OH)D3 concentration (IRR = 0.89; 95% CI, 0.80 to 0.98). Similarly, number of symptoms in the ADHD subscales decreased in relation to higher maternal 25(OH)D3 concentration (IRR per 10-ng/mL increment = 0.89; 95% CI, 0.79 to 0.99 for inattention scale; and IRR = 0.88; 95% CI, 0.78 to 0.99 for hyperactivity-impulsivity scale). Using diagnostic criteria, researchers found that association of increasing maternal 25(OH)D3 with a lower risk of ADHD DSM-IV (relative risk ratio per 10-ng/mL increment = 0.87; 95% CI, 0.72, 1.06) and ICD-10 hyperkinetic disorder (relative risk ratio = 0.72; 95% CI, 0.49, 1.04) in children.			
			Zhu et al. (2015)	Cohort	China, 2008	Children aged 16–18 months	*n* = 363	Certified examiners; BSID	Toddlers in lowest quintile of cord blood 25(OH)D exhibited a deficit of 7.60 (95% CI, 212.4 to 22.82; *p* = 0.002) and 8.04 (95% CI, 212.9 to 23.11; *p* = 0.001) points in the MDI and PDI scores, respectively, compared with the reference category. Mean 25(OH)D (range): 4.9 (2.2–44.4).			
			Chen et al. (2016)	Case-control	China, January 2014–December 2015	Mothers (1st trimester) 25(OH)D; children aged 3–7 years	*n* = 136 (68/68)	DSM-V	Mothers in autistic group had significantly lower maternal serum levels of 25(OH) D than in typically developing group [19.2 (IQR: 15.8–22.9) ng/mL vs. 24.3 (19.3–27.3) ng/mL, *p* < 0.001] with 55.9% and 29.4% being vitamin D deficient, respectively (*p* < 0.001). Levels of 25(OH) D increased with decreasing severity of ASD as defined by CARS score (*r* = −0.302, *p* < 0.001). Maternal first-trimester serum levels of 25(OH)D in the lower 3 quartiles (quartile 1, 2, 3) (compared to the highest quartile) were associated with increased odds of ASD diagnosis in offspring [OR (95% CI) Q1: 1.36 (0.84 to 2.58, *p* = 0.25); Q2: 2.68 (1.44 to 4.29, *p* = 0.006); Q3: 3.99 (2.58 to 7.12, *p* < 0.001)].			
			Magnusson et al. (2016)	Cohort	Sweden, 2001–2011	Stockholm Youth Cohort; Swedish-born individuals aged 4–17 years (no control)	*N* = 509,639 (9882 ASD cases; 2476 with intellectual disability, 7406 without intellectual disability)	National and regional registers; ASD diagnosis	Maternal vitamin D deficiency was associated with offspring risk of ASD with, but not without, intellectual disability (aORs 2.51; 95% CI, 1.22 to 5.16 and 1.28, 95% CI, 0.68 to 2.42).			
			Darling et al. (2017)	Cohort	UK, 1991–1992	Mothers (29 weeks) 25(OH)D; children aged 6–42 months; 7–9 years	*n* = 7065	Maternal report; SDQ	Children of vitamin D–deficient mothers (<50.0 nmol/L) were more likely to have scores in the lowest quartile for gross motor development at 30 months (OR 1.20; 95% CI, 1.03 to 1.40), fine-motor development at 30 months (OR 1.23; 95% CI, 1.05 to 1.44), and social development at 42 months (OR 1.20; 95% CI; 1.01 to 1.41) than vitamin D–sufficient mothers (≥50.0 nmol/L). Median (IQR) 25(OH)D: 24.5 (17.2–33.9).			
			Chawla et al. (2017)	Prospective	U.S., 2009–2011	Newborn Epigenetic Study; mothers who measured 25(OH)D concn in plasma samples in first or second trimester; children aged 12–24 months	*n* = 218 mother–infant pairs	Maternal report; ITSEA	Black mothers had much lower 25(OH)D concentrations than white and Hispanic mothers.			
			Gould et al. (2017)	RCT of DHA supplementation in pregnancy	Australia; 2005–2008	Children aged 18 months–4 years	299–323	Psychologist report; BSID-III	25(OH)D was not associated with cognitive, motor, social-emotional or adaptive behavior scores at 18 months or cognitive score at age 4 years.			
			Laird et al. (2017)	Cohort	Republic of Seychelles, 2001	Mothers 25(OH)D; children aged 5 years	*n* = 189	CBCL; total *t* score	Maternal 25(OH)D concentrations were not associated with child anthropometric or neurodevelopmental outcomes. Mean (SD) 25(OH)D: 22.4 (11.4) ng/ml			
			Mossin et al. 2017)	Cohort	Denmark, 2010–2012	Newborns (cord blood) 25(OH)D; children aged 1.5–5 y	*n* = 1233	Parental report; CBCL; ADHD score	Cord blood 25(OH)D levels > 25 nmol/L and >30 nmol/L were associated with lower attention deficit hyperactivity disorder scores than levels = 25 nmol/L or <25 nmol/L (*p* = 0.035) and =30 nmol/L or <30 nmol/L (*p* = 0.043), respectively.			
			Daraki et al. (2018)	Cohort	Greece, 2006–2007	Mothers (13 weeks) 25(OH)D; children aged 4 y	*n* = 487	SDQ	Children of mothers with high 25(OH)D levels had also fewer total behavioral difficulties (β-coeff: −1.25, 95% CI, −2.32, to –0.19) and externalizing symptoms (β-coeff: −0.87, 95% CI to −1.58, −0.15) at preschool age.			
			Vinkhuyzen et al. (2017)	Cohort	Netherlands, April 2002 and January 2006	Embedded in the Generation R Study; mothers (mid-gestation) 25(OH)D; children aged 6 y	*n* = 3895, *n* = 2870	Clinical records; ASD diagnosis	Individuals in the 25(OH)D-deficient group at mid-gestation had more than twofold increased risk of ASD (OR = 2.42, 95% CI, 1.09 to 5.07, *p* = 0.03) compared with the 25(OH)D-sufficient group.			
			Vinkhuyzen et al. (2018)	Prospective cohort	Netherlands, April 2002 and January 2006	Embedded in the Generation R Study; mothers (mid gestation) 25(OH)D; children aged 6 y	*n* = 4229	Parental report; SRS: autism-related traits	Compared with the 25OHD sufficient group (25OHD >50 nmol/L), those who were 25(OH)D deficient had significantly higher (more abnormal) SRS scores (mid- gestation *n* = 2866, β = 0.06, *p* < 0.001; cord blood *n* = 1712, β = 0.03, *p* = 0.01).			
			Wang et al. (2018)	Cohort	China, 2012–2013	Newborns (cord blood) 25(OH)D; children aged 2 y	*n* = 552	Parental report; ASQ: gross and fine-motor skills	Median of the 25(OH)D concentration in cord blood was 22.4 ng/mL (IQR, 27.3–8.6). Infants born in winter had lower 25(OH)D concentration. 25(OH)D deficiency was not associated with weight *z*-score (mean difference, 0.07; 95% CI, −0.09 to 0.23), length *z*-score (mean difference, 0.01; 95% CI, −0.19 to 0.21), head circumference *z*-score (mean difference, −0.06; 95% CI, −0.27 to 0.15) and BMI *z*-score (mean difference, 0.09; 95% CI, −0.07 to 0.25) or neurodevelopment during infancy, adjusting for sex, socioeconomic position, prepregnancy maternal BMI, and maternal and neonatal characteristics.			
			Veena et al. (2017)	Cohort	India, 1997–1998	Mothers (30 weeks) 25(OH)D; children aged 9–10 and 13–14 y	*n* = 468; *n* = 472	Psychologist report; KABC	No evidence of an association between maternal vitamin D status and offspring cognitive function.			
			Tylavsky et al. (2015)	Cohort	U.S., 2006–2011	CANDLE Study Mothers (2nd trimester) 25(OH)D; children aged 2 years	*n* = 1020 mother; 16–28 weeks of gestation	BSID	Gestational 25(OH)D was positively associated with cognitive scaled scores, receptive language, and expressive language (*p* < 0.001) Mean (range) 25(OH)D: 22.3 (5.9–68.4) ng/ml			
			Gale et al. (2008)	Cohort	UK (Southampton), 1991–1992	Mothers (32 weeks) 25(OH)D; children aged 9 y	*n* = 178	Mother report; SDQ	No statistically significant associations between maternal 25(OH)D concn and full-scale, verbal or performance IQ, assessed by the Wechsler Abbreviated Scale of Intelligence (*p* > 0.005) Median (IQR) 25(OH)D:25 (15–30.1) ng/ml			
			Hanieh et al. (2014)	Cohort	Vietnam, 2010–2012	Mothers (32 weeks) 25(OH)D; children aged 6 months	*n* = 960	Psychologist report; BSID	Infants born to women with 25(OH)D deficiency (<37.5 nmol/L) had lower developmental language scores than infants born to women who were vitamin D replete (≥75 nmol/L) (mean difference, −3.48; 95% CI, −5.67 to –1.28).			
			Whitehouse et al. (2012)	Cohort	Perth, Western Australia, 1989–1991	Mothers (18 weeks) 25(OH)D; children aged 2–17 y	412–652	Parental report; CBCL: total behavior, internalizing behavior, externalizing behavior	No significant associations between maternal 25(OH)D serum quartiles and offspring behavioral/emotional problems at any age.			
			Whitehouse et al. (2013)	Cohort	Australia, May 1989 and Nov. 1991	Raine Study Mothers gestational age 16–20 wks; children aged 5–17 y	*n* = 406	Parental report; ASD diagnosis; autism spectrum quotient	Offspring of mothers with low 25(OH)D concentrations (<49 nmol/L) were at increased risk for “high” scores (≥2 SD above mean) on the Attention Switching subscale (OR, 5.46; 95% CI, 1.29 to 23.05).			
			Strøm et al. (2014)	Cohort	Denmark, 1988–1989	Mothers (>30 weeks) 25(OH)D; children aged 22 y	*n* = 850	Population-based registry; prescription for medication or hospital admission for: ADHD	Direct association between maternal vitamin D status and offspring depression (*p*_trend_ = 0.01).			
			Gustafsson et al. (2015)	Cohort	Sweden, 1978–2000	Newborns (cord blood) 25(OH)D3; children aged 5–17 y	*n* = 404 (202/202)	DSM-III-R used before 1994 and DSM-IV used after 1994	No differences in cord blood vitamin D concentration were found between children with ADHD (median 13.0 ng/mL) and controls (median 13.5 ng/mL) (*p* = 0.43).			
			Keim et al. (2014)	Case-control	U.S., 1959–1965	Collaborative Perinatal Project; *n* = 55,000 pregnant women; children aged 8 month and 4–7 y	3146–3587	Psychologist report; BSID	IQ at age 7 was associated with both maternal and cord blood 25(OH)D (β for 5-nmol/L increment of maternal 25(OH)D = 0.10; 95% CI, 0.00 to 0.19).			

ASD, autism spectrum disorder; RCT, randomized controlled trial; 95% CI, 95% confidence interval; ABC ^1^, Autism Behavior Checklist; ABC ^2^, Aberrant Behaviour Checklist; ADHD, Attention deficit–hyperactivity disorder; ADOS, Autism Diagnostic Observation Schedule; aOR, Adjusted odds ratio; ASQ, Ages and Stages Questionnaire; BMI, body mass index (kilograms per square meter of body surface area); BSID, Bayley Scales of Infant Development; CARS, Childhood Autism Rating Scale; CBCL, Child Behavior Checklist; DSM, Diagnostic and Statistical Manual; GDNF, glial cell line–derived neurotrophic factor; IQR, interquartile range; IRR, incidence rate ratio; ITSEA, Infant–Toddler Social and Emotional Assessment; KABC, Kaufmann Assessment Battery for Children; anti-MAG, anti- myelin-associated glycoprotein; DHA, docosahexaenoic acid; MCHAT, Modified Checklist for Autism in Toddlers; MDI, mental development index; NGF, nerve growth factor; OR, odds ratio; PDDBI, Pervasive Developmental Disorder Behavior Inventory; PDI, Psychomotor development index; SDQ, Strengths and Difficulties Questionnaire; SD, standard deviation; SRS, Social Responsiveness Scale.
